# Methotrexate inhibits effects of platelet-derived growth factor and interleukin-1β on rheumatoid arthritis fibroblast-like synoviocytes

**DOI:** 10.1186/s13075-018-1554-7

**Published:** 2018-03-20

**Authors:** Beatrice Bergström, Hans Carlsten, Anna-Karin Hultgård Ekwall

**Affiliations:** 1 Department of Rheumatology and Inflammation Research, Institute of Medicine, The Sahlgrenska Academy, University of Gothenburg, Box 480, 405 30 Gothenburg, Sweden; 2 Centre for Bone and Arthritis Research, University of Gothenburg, Gothenburg, Sweden

**Keywords:** Rheumatoid arthritis, Fibroblast-like synoviocytes, Cell cycle, Platelet-derived growth factor, Interleukin-1beta, Methotrexate

## Abstract

**Background:**

A key feature of joints in rheumatoid arthritis (RA) is the formation of hyperplastic destructive pannus tissue, which is orchestrated by activated fibroblast-like synoviocytes (FLS). We have demonstrated that the RA risk gene and tumor suppressor Limb bud and heart development (LBH) regulates cell cycle progression in FLS. Methotrexate (MTX) is the first-line treatment for RA, but its mechanisms of action remain incompletely understood. Here, we studied the effects of MTX on mitogen-induced FLS proliferation and expression of cell cycle regulators in vitro.

**Methods:**

Primary FLS from patients with RA or osteoarthritis were stimulated with the mitogen platelet-derived growth factor (PDGF) and the cytokine interleukin-1β (IL-1β) in the presence or absence of MTX. Cells were then subjected to qPCR for gene expression and cell cycle analysis by flow cytometry.

**Results:**

Stimulation with PDGF and IL-1β increased the percentage of FLS in the G2/M phase and shifted the cell morphology to a dendritic shape. These effects were inhibited by MTX. Furthermore, PDGF + IL-1β reduced *LBH* mRNA expression. However, MTX treatment yielded significantly higher transcript levels of *LBH*, and of *CDKN1A* (p21) and *TP53* (p53), compared to untreated samples upon mitogen stimulation. The expression of DNA methyltransferase-1 (*DNMT1*) was also higher in the presence of MTX and there was strong correlation between *DNMT1* and *LBH* expression.

**Conclusions:**

Therapeutic concentrations of MTX abolish the effects of PDGF and IL-1β on tumor suppressor expression and inhibit mitogen-promoted FLS proliferation. These data demonstrate novel and important effects of MTX on pathogenic effector cells in the joint, which might involve epigenetic mechanisms.

## Background

The rheumatoid arthritis (RA) joint pathology is characterized by persistent inflammation and synovial hyperplasia, leading to destruction of cartilage and bone and ultimately functional disability [1]. Key players in these processes are fibroblast-like synoviocytes (FLS), which develop an aggressive, tumor-like phenotype [2] with increased invasiveness and disturbed control of cell proliferation [3]. The local FLS expansion in the synovial lining layer correlates with clinical disease activity parameters in RA [4]. Also, treatment with cell cycle inhibitors targeting FLS ameliorates arthritis in mouse models [5, 6]. Thus, studies of pathways regulating the proliferation of FLS are of considerable interest.

FLS in RA are activated to an aggressive phenotype by repeated exposure to numerous pro-inflammatory cytokines and growth factors acting on top of predisposing genetic and epigenetic factors [7, 8]. Stimulating FLS in vitro with tumor necrosis factor (TNF) and interleukin-1β (IL-1β) induces a variable expression of many of the genes characterizing the aggressive RA FLS phenotype. Platelet-derived growth factor (PDGF) is a known fibroblast mitogen and both PDGF and IL-1β have been reported to increase FLS proliferation [9, 10]. Interestingly, the cytokine (TNF or IL-1β) potentiates the effects of growth factors (PDGF and transforming growth factor (TGF)-β) on cytokine and matrix metalloproteinase production in FLS [11].

Moreover, RA FLS display intrinsic dysregulation of proliferation and apoptosis pathways, such as abnormalities of the tumor suppressor p53 [12] and its downstream mediator p21 [13] which in the normal synoviocyte induces cell cycle arrest. The number of p21-positive FLS inversely correlates with the thickness synovial lining layer in RA [13]. Recently, the transcription regulator, Limb bud and heart development (LBH), was identified as a new candidate gene for the pathogenic FLS phenotype in RA [14]. Reduced expression of this tumor suppressor in FLS has been suggested to increase the risk of RA development. LBH is regulated by growth factors and epigenetic mechanisms [15] and modulates proliferation in primary FLS by regulating cell cycle progression at the G1 to S phase transition checkpoint [16, 17]. In particular, a regulatory DNA element in the *LBH* gene region is hypo-methylated in RA. This DNA element is believed to negatively regulate *LBH* transcription.

Methotrexate (MTX) is still the first-line treatment for RA, but its exact mechanisms of action and target cells remain incompletely understood. The anti-inflammatory effect of low-dose MTX used in rheumatic diseases has in part been attributed to release of adenosine [18]. Studies have also demonstrated that MTX treatment restores global DNA methylation of peripheral blood mononuclear cells [19] and increases expression of cell cycle checkpoint genes in the T cells in patients with RA [20]. However, the effects of MTX on FLS in RA are largely unknown.

In order to further understand the pharmacodynamics of MTX in RA and to identify novel drug targets and markers to predict drug response, this study aimed to investigate the effects of MTX on mitogen-induced FLS proliferation in vitro, and in particular on the expression of cell cycle regulators.

## Methods

### Synovial samples

Human synovial tissue specimens were obtained from patients with RA (*n* = 6) or osteoarthritis (OA) (*n* = 7) during joint replacement surgery at Sahlgrenska University Hospital in Sweden. All Patients with RA fulfilled the American College of Rheumatology 1987 revised criteria for the disease [21] and all had established seropositive and erosive disease. MTX was stopped ≥ 2 weeks before surgery. The procedures were approved by the Ethics Committee of Gothenburg, and all patients gave written informed consent.

### Cell culture

Primary FLS were isolated as previously described [2] and cultured in Dulbecco’s modified Eagle’s medium (DMEM) GlutaMAX (Gibco, Carlsbad, CA, USA) supplemented with antibiotics (penicillin/streptomycin, gentamicin) and 10% heat-inactivated fetal bovine serum (FBS), in a humidified 5% CO_2_ atmosphere. Cells at passages three to nine were seeded into 6-well or 24-well plates for experiments. Stimulation with the human recombinant proteins PDGF-BB (Invitrogen, Carlsbad, CA, USA) and IL-1β (R&D Systems, Minneapolis, MN, USA) was performed in 1% FBS medium after overnight serum starvation.

### MTX dose and treatment protocol

Therapeutic doses of MTX range from 10 to 25 mg weekly among patients with RA, resulting in peak serum concentrations at 1 μM [22, 23]. Concentrations are higher in synovial tissue than in blood [24]. In previous in vitro studies of FLS, concentrations of MTX up to 100 μM have been used [25]. Treatment of FLS cultures for 48 h with 0.01–10 μM MTX had no effect on viability or apoptosis of FLS in RA [26].

Here, FLS were pretreated for 24 h with MTX (Apoteket, Sweden) or control medium, then stimulated with PDGF + IL-1β in 1% FBS medium in the presence or absence of MTX. The cells were harvested 24–48 h after mitogen stimulation for gene expression or cell cycle analysis. Only FLS lines responding to mitogen stimulation (defined by a fold change in *LBH* mRNA below 0.3 compared to the unstimulated control) under the present experimental time frame were included.

### Cell cycle analysis

**Fig. 1:**
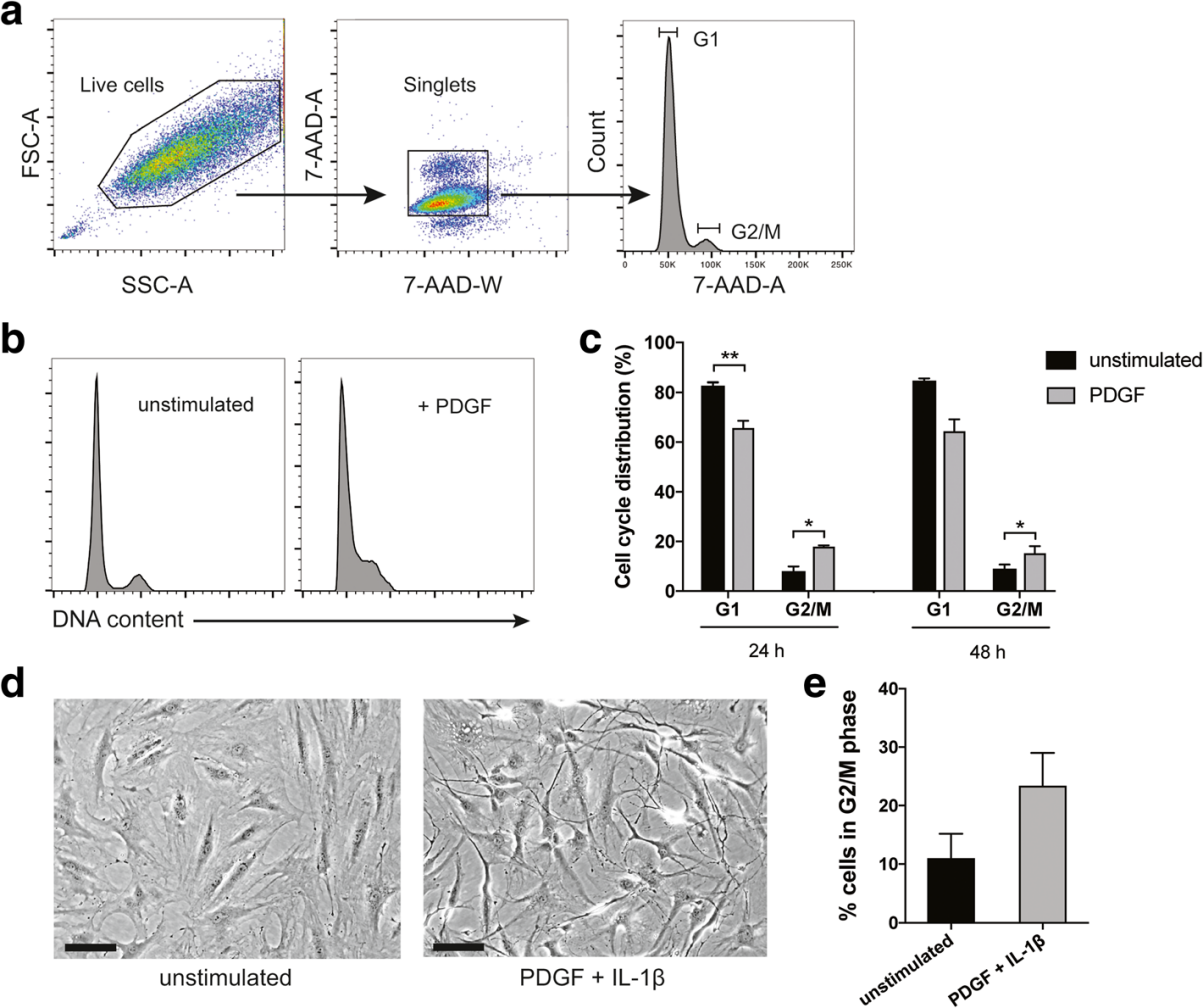
Effects of mitogens on the cell cycle and cell morphology of fibroblast-like synoviocytes (FLS). Primary rheumatoid arthritis (RA) FLS were serum-starved and stimulated with platelet-derived growth factor (PDGF) or a combination of PDGF and IL-1β. The cells were then harvested and DNA-stained followed by flow cytometry. **a** Gating strategy identifying live cells and singlets and histogram gating of cell cycle analysis by DNA content. **b** Representative histograms of FLS in an unstimulated state and upon PDGF stimulation. **c** Percentage of cells in the G1 and G2/M phase, respectively, in unstimulated FLS and upon stimulation with PDGF (20 ng/mL) for 24 and 48 h, respectively. **d** Morphology of FLS in 2D culture, before stimulation and after incubation for 48 h with PDGF (20 ng/mL) + IL-1β (2 ng/mL), as visualized by light microscopy. Scale bar 20 μM. **e** Percentage of cells in the G2/M phase upon PDGF + IL-1β stimulation compared to percentage of cells without stimulation. Values are the mean ± SEM of three different FLS lines. *p < 0.05, **p < 0.01. FSC-A, forward scatter-area; SSC-A, side scatter-area

FLS were detached by trypsin-EDTA and resuspended in PBS, fixed in 70% ethanol and stained with the DNA label 7-amino-actinomycin D (7-AAD) (BD Pharmingen, San Diego, CA, USA). DNA content was measured using a FACSVerse flow cytometer and FACSuite software (BD Biosciences) on at least 10,000 cells/sample. Data were analyzed using FlowJo software (Tree Star, Ashland, OR, USA) and the percentages of cells in G1 and G2/M phase, respectively, were calculated from histograms of DNA content after exclusion of debris and doublets. The gating strategy is presented in Fig. 1a.

### RNA extraction and gene expression analysis

Total RNA was isolated from cells using RNeasy Mini Kit (Qiagen) and quantified with a NanoDrop 100 spectrophotometer (Thermo Scientific). Complementary DNA was synthesized using TaqMan reverse transcription agents (Applied Biosystems, Carlsbad, CA, USA) with 250 ng RNA per reaction. Quantitative polymerase chain reaction (qPCR) was performed on a ViiA Real-Time PCR System using TaqMan Gene Expression Assays (Applied Biosystems) for *GAPDH* (Hs99999905_m1), *LBH* (Hs00368853_m1), *CDKN1A* (Hs00355782_m1), *CDKN2A* (Hs00923894_m1), *TP53* (Hs01034249_m1), *CCND1* (Hs00765553_m1), *CCNE1* (Hs01026536_m1), *CDC25A* (Hs00947994_m1), *DNMT1* (Hs00154749_m1), and *DNMT3A* (Hs01027166_m1). Cycle threshold (Ct) values were normalized to *GAPDH.* Results were presented as fold changes calculated by the 2^-ΔΔCt^ method.

### Statistical analysis

Results are presented as the mean ± SEM. Differences were evaluated by paired Student’s *t* test for comparison of two groups, or one-way analysis of variance (ANOVA) followed by Tukey’s post hoc test for comparison of multiple groups. Shapiro-Wilk’s normality test was used to assess sample distribution. Statistical analysis of differences in gene expression was performed on ΔCt values. Correlation between fold changes in gene expression was tested with Spearman’s rank correlation coefficient. The analyses were performed using GraphPad Prism software (version 7.0b; GraphPad Inc., La Jolla, CA, USA): *p* values less than 0.05 were considered significant.

## Results

### PDGF and IL-1β induce FLS proliferation

It has previously been reported that IL-1β enhances FLS proliferation in vitro as measured by ^3^H-thymidine incorporation [10], and that PDGF induces colony growth of primary FLS [9]. In this study, we used cell cycle analysis by flow cytometry to assay the effects of the mitogen on cultured RA FLS. Stimulation with PDGF 20 ng/mL alone promoted cell cycle progression as expected (Fig. 1b and c), increasing the percentage of cells in the G2/M phase (18 ± 1% versus 8 ± 2% in unstimulated controls, *p* = 0.03) while reducing the percentage of cells in the G1 phase (66 ± 3% versus 83 ± 1% in unstimulated controls, *p* = 0.009), after 24 h. Similar results were observed after 48 h (Fig. 1c). Upon stimulation with a combination of PDGF 20 ng/mL and IL-1β 2 ng/mL, a pronounced shift in FLS morphology to a more dendritic cell shape was seen, with cells extending long pseudopodia (Fig. 1d). The mean percentage of cells in the G2/M phase was also higher with this combination compared to unstimulated cells (Fig. 1e), although not statistically significant (23 ± 6% of cells in the G2/M phase versus 11 ± 4% in unstimulated controls, *p* = 0.14).

### MTX inhibits the effects of mitogens on FLS proliferation

**Fig. 2:**
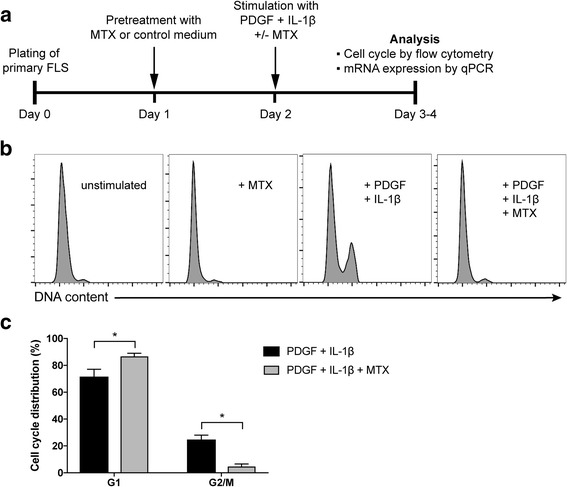
Effects of methotrexate (MTX) on mitogen-induced cell cycle progression in fibroblast-like synoviocytes (FLS). **a** Design and timeline of the current MTX experiments. **b** Rheumatoid arthritis (RA) FLS were stimulated with platelet-derived growth factor (PDGF, 20 ng/mL) + IL-1β (2 ng/mL) for 24 h in the presence or absence of 1 μM MTX, or treated with MTX only. The cells were then DNA-stained followed by cell cycle analysis by flow cytometry. Representative histograms of DNA content with indicated treatments are shown. **c** Percentage of cells in the G1 and G2/M phase, respectively, in three different FLS lines. The experiments were repeated more than three times. Values are mean ± SEM. *p < 0.05. OA, osteoarthritis

To investigate the effects of MTX on the proliferation and gene expression of RA FLS in an inflammatory setting, we developed an experimental set-up as overviewed in Fig. 2a. In cell cycle analysis by flow cytometry, MTX treatment alone did not affect the DNA content distribution seen in unstimulated cultures (Fig. 2b). Stimulation with PDGF + IL-1β could induce cell cycle progression, but this effect was abolished by the presence of MTX. Treatment with MTX resulted in only 5 ± 2% of the cell population in the G2/M phase upon mitogen stimulation, compared to 25 ± 4% on mitogen stimulation alone (*p* = 0.02) (Fig. 2c). Instead, cells were halted in the G1 phase when MTX was present upon mitogen stimulation (87 ± 3% versus 71 ± 6% in the absence of MTX, *p* = 0.04) (Fig. 2c).

### PDGF and IL-1β reduce *LBH* expression

**Fig. 3:**
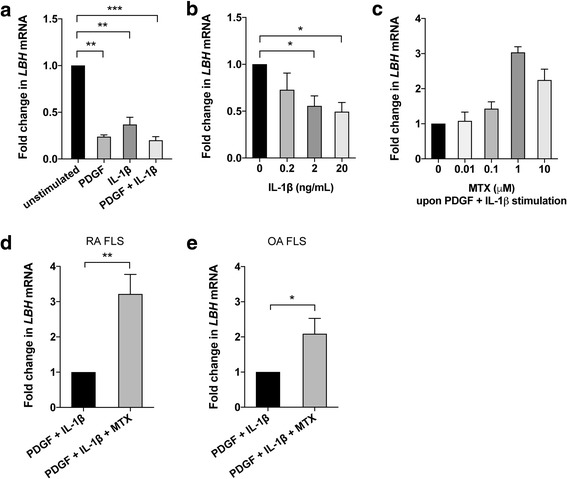
*LBH* expression in fibroblast-like synoviocytes (FLS) in response to platelet-derived growth factor (PDGF) and IL-1β, with or without methotrexate (MTX). **a, b** Fold change in mRNA levels of *LBH* compared to unstimulated was assessed by qPCR in serum-starved FLS after stimulation for 12 h with PDGF (10 ng/mL) and/or IL-1β (2 ng/mL) (**a**), or IL-1β at different concentrations (0.2–20 ng/mL) (**b**). **c** Fold change in *LBH* mRNA levels was measured after 48 h of mitogen stimulation (PDGF 20 ng/mL + IL-1β 2 ng/mL) in the presence or absence of MTX at various doses (0.01–10 μM; *n* = 2). **d, e** Treatment with 1 μM MTX upon PDGF 20 ng/mL + IL-1β 2 ng/mL stimulation was evaluated in rheumatoid arthritis (RA) FLS (**d**) and in osteoarthritis (OA) FLS (**e**). Values are the mean fold change ± SEM of three to six different FLS lines. *p < 0.05, **p < 0.01, ***p < 0.0001

It has recently been demonstrated that LBH is a modulator of FLS proliferation and that it is regulated by growth factors implicated in RA. PDGF has been shown to reduce *LBH* expression in a dose-dependent manner and with a peak effect at 12 h [16]. We found that stimulation with IL-1β also reduced *LBH* mRNA expression in FLS and to the same extent as with PDGF (Fig. 3a). Significant effects of IL-1β were obtained at 2 ng/mL and 20 ng/mL (Fig. 3b). The combination of PDGF + IL-1β yielded the strongest effect on *LBH* expression (Fig. 3a) and was therefore employed in subsequent experiments.

### MTX inhibits the effects of mitogens on *LBH* expression

Next, we examined if MTX inhibited the effects of PDGF + IL-1β on *LBH* expression in FLS. In the presence of MTX, there was a dose-dependent increase in *LBH* mRNA levels after mitogen stimulation compared to cultures without MTX. The optimal concentration was found to be 1 μM MTX (Fig. 3c), which corresponds to concentrations found in patients treated with therapeutic doses of the drug (see “Methods”). *LBH* expression was increased 3.2 ± 0.5 fold (*p* = 0.002) after MTX treatment in RA FLS (Fig. 3d). Since osteoarthritis (OA) FLS also can be “activated” to some extent under certain conditions in vitro [27], we repeated the experiment with OA samples. We found that stimulation with PDGF + IL-1β reduced *LBH* expression and MTX treatment increased the expression 2.2 ± 0.5 fold (*p* = 0.02) in OA FLS (Fig. 3e). The difference between RA and OA FLS was not statistically significant under the present conditions.

### MTX abolishes the effects of mitogens on *CDKN1A* and *TP53* expression

**Fig. 4:**
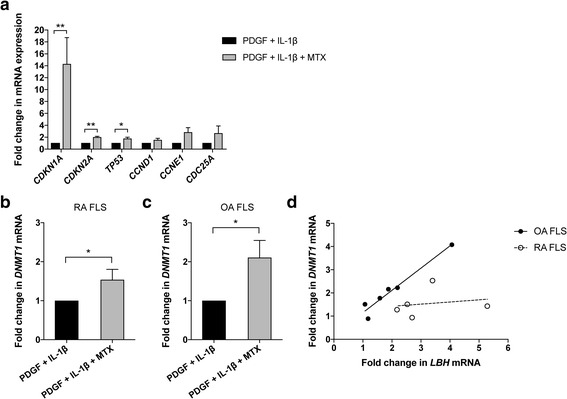
Expression of cell cycle regulator genes and *DNMT1* in response to mitogens +/− methotrexate (MTX). Treatment of fibroblast-like synoviocytes (FLS) was performed according to the protocol in Fig. 2a (platelet-derived growth factor (PDGF) 20 ng/mL + IL-1β 2 ng/mL +/− MTX 1 μM). mRNA expression was analyzed by qPCR after 48 h of mitogen stimulation. **a** Fold change in mRNA levels of the indicated cell cycle regulator genes. **b**, **c** Fold change in *DNMT1* expression in rheumatoid arthritis (RA) FLS (**b**) and in osteoarthritis (OA) FLS (**c**). Values are the mean fold change ± SEM in three to six different FLS lines. *p < 0.05, **p < 0.01. **d** Correlation between *DNMT1* and *LBH* expression in OA FLS (solid circles; *n* = 6) and RA FLS (open circles; *n* = 5). Each dot represents an individual FLS line. Values are fold change in mRNA expression on PDGF + IL-1β + MTX treatment compared to PDGF + IL-1β alone. Lines show fitting of data to a linear regression

We continued to study the effects of MTX on other cell cycle regulator genes in RA FLS, as presented in Fig. 4a. *CDKN1A* encodes the protein p21 (CIP1/WAF1), which regulates cell cycle progression primarily by acting as a cyclin-dependent kinase inhibitor (CDKI) at the G1 to S phase transition*.* Strikingly, the *CDKN1A* mRNA expression was 14.3 ± 4.4 fold higher (*p* = 0.006) in the presence of MTX upon mitogen stimulation compared to without MTX. Another CDKI, p16 encoded by the *CDKN2A* gene, was also significantly increased (2.0 ± 0.1 fold, *p* = 0.002). One of the regulators of p21 is the transcription factor p53 (encoded by *TP53*), known to inhibit cell cycle activity and inflammation. The mRNA levels of *TP53* were 1.8 ± 0.2 fold higher (*p* = 0.03) in the presence of MTX. Within the experimental time frame, we did not detect any significant effects on the transcription of *CCND1* (cyclin D1), *CCNE1* (cyclin E1) or *CDC25A* (M-phase inducer phosphatase 1).

### *DNMT1* expression is increased after MTX treatment and correlates with *LBH*

It has been demonstrated that IL-1β reduces the expression of DNA methylating enzymes and global DNA methylation in FLS [28]. Since MTX has previously been reported to alter DNA methylation in lymphocytes and monocytes in patients with RA, we also assessed the expression of DNA methyltransferases (DNMTs) in FLS in our experimental setting. Stimulation with PDGF + IL-1β reduced *DNMT1* mRNA expression, confirming earlier reports. However, in the presence of MTX the *DNMT1* transcript levels were significantly higher compared to mitogen-stimulated FLS without MTX treatment, both in RA FLS (1.5 ± 0.3 fold, *p* = 0.04) (Fig. 4b) and OA FLS (2.1 ± 0.4 fold, *p* = 0.03) (Fig. 4c). There were no significant differences in *DNMT3A* expression (data not shown). Recent data suggest that the expression of *LBH* is in part regulated by DNA methylation in FLS [15]. We found strong correlation between fold change in *DNMT1* and *LBH* mRNA expression in the OA samples (Spearman’s correlation coefficient, *r*_*s*_ = 0.94, *p* = 0.017) but not in RA FLS (*r*_*s*_ = 0.30, *p* = 0.68). Interestingly, the RA and OA samples clustered in distinct groups (Fig. 4d).

## Discussion

A hallmark of the pathogenesis of RA is the formation of destructive pannus tissue in which activated FLS play a key role. This hyperplastic invasive tissue is overpopulated by FLS mediating persistent inflammation and joint destruction by production of pro-inflammatory cytokines, chemokines, growth factors and matrix degrading enzymes. Both increased resistance to apoptosis and increased proliferation is believed to cause the increased FLS number [8] and the predominant mechanism most probably varies by disease duration and between individuals. Targeting cytokines and immune cells in the treatment of RA has proven insufficient in a substantial number of patients and there is a need for more efficient treatment. Studies of the mechanisms regulating FLS proliferation are therefore of utter importance, as there is currently no therapy specifically targeting these pivotal effector cells in the joint in RA.

Low-dose MTX is the standard of care in the treatment of RA. About one third of the patients will reach low disease activity with MTX mono-therapy and another third will reach good clinical response in combination with a biologic agent [29]. The prevailing theory of the pharmacodynamic effects of MTX is that it targets the activity of immune cells. However, a recent study demonstrates the inhibitory effect of MTX on TNF-induced NF-κB activation in FLS *in vitro* [30].

We performed *in vitro* studies where the proliferative FLS phenotype was induced by PDGF + IL-1β. This is to our knowledge the only study investigating effects on cell cycle progression of FLS with this combination. IL-1β has been shown to reduce expression of DNA methylating enzymes (DNMTs) in FLS [28] and might thus act on a different regulatory level (epigenetic) than other cytokines and growth factors and could therefore potentiate or modulate their effects on gene expression. Interestingly, we found that MTX treatment inhibited cell cycle progression in PDGF + IL-1β-stimulated FLS, which suggests a new mechanism of action of this drug.

Furthermore, we studied the effects of MTX on the recently identified RA risk gene, *LBH*, which encodes a highly conserved transcription regulator and the expression of LBH in FLS is believed to be protective in RA [31]. LBH has been shown to regulate FLS growth by acting as a tumor suppressor [16]. A recent study demonstrating that LBH deficiency exacerbates arthritis in an *in vivo* model supports these findings [17]. In our experiments, the mitogens PDGF and IL-1β were found to reduce *LBH* mRNA expression in RA FLS, and MTX treatment abrogated these effects. A pronounced effect of MTX was also seen on the cell cycle checkpoint gene *CDKN1A,* encoding p21, and to some extent on *CDKN2A* (p16) and *TP53* (p53). This is consistent with earlier studies of T cells in RA, where MTX treatment induces the expression of p21 and p53 [20].

In this and earlier studies, PDGF also reduced *LBH* expression in OA FLS [16] and we demonstrated that MTX can increase expression of *LBH* and other tumor suppressor genes after mitogen stimulation not only in RA FLS but also to some extent in OA FLS. The gene expression profiles of primary FLS from patients with OA converge with those of RA FLS under high serum conditions *in vitro* [27]*.* This finding suggests some common intrinsic response patterns of FLS, and synovitis is common in OA [32]. However, sustained exposure to growth factors and cytokines, as in the joints in RA, has been demonstrated to prime FLS to enhanced pathogenic responses [33]. This together with genetic and epigenetic factors of RA FLS [8] might explain the differences in the degree of synovial activation and inflammation in the two diseases. Interestingly, treatment with anti-TNF agents has failed to provide symptomatic benefits in OA trials [34] but a recent study also suggests analgesic efficacy of MTX in OA [35]. It is important to point out that the RA synovial samples in this study were obtained from patients with end-stage disease and it is possible that the differences in results between RA and OA FLS would have been larger in FLS from early RA.

We have earlier demonstrated that *LBH* expression in FLS is regulated by DNA methylation [15]. Since a suggested mechanism of action of MTX in lymphocytes is interference with DNA methylation, we also studied the effects of MTX on DNA methylating enzymes in FLS. Indeed, we found that the mRNA expression of *DNMT1* was higher after MTX treatment compared to untreated PDGF + IL1β-stimulated FLS. Moreover, there was strong correlation between *DNMT1* and *LBH* expression in MTX-treated OA FLS, suggesting that MTX might mediate the effects on cell cycle regulator gene expression in FLS by restoring epigenetic patterns. In RA FLS, the samples clustered with high fold change in *LBH* expression, but the correlation with *DNMT1* expression was not present, indicating that other factors also influence gene expression. This is in agreement with our previous findings that *LBH* expression in RA FLS is regulated by growth factors as well as by DNA methylation and genetic polymorphism of a regulatory element [15].

## Conclusion

We demonstrate that therapeutic concentrations of MTX abolish mitogen-promoted proliferation of RA FLS and inhibit the effects of mitogens on cell cycle regulators. This could be one hitherto unknown mode of action contributing to the efficacy of MTX in RA. Understanding the molecular mechanisms of the effects of MTX on FLS is of great importance for the development of novel, more FLS-specific drugs and for identifying biomarkers to predict response to MTX.

## Abbreviations

7-AAD: 7-Amino-actinomycin D
ANOVA: Analysis of variance
CDKI: Cyclin-dependent kinase inhibitor
DNMT: DNA methyltransferase
FBS: Fetal bovine serum
FLS: Fibroblast-like synoviocytes
IL: Interleukin
LBH: Limb bud and heart development
MTX: Methotrexate
OA: Osteoarthritis
PBS: Phosphate-buffered saline
PDGF: Platelet-derived growth factor
qPCR: Quantitative polymerase chain reaction
RA: Rheumatoid arthritis
TGF: Transforming growth factor
TNF: Tumor necrosis factor
